# Novel Chitohexaose Analog Protects Young and Aged mice from CLP Induced Polymicrobial Sepsis

**DOI:** 10.1038/s41598-019-38731-3

**Published:** 2019-02-27

**Authors:** Pragnya Das, Santosh K. Panda, Beamon Agarwal, Sumita Behera, Syed M. Ali, Mark E. Pulse, Joseph S. Solomkin, Steven M. Opal, Vineet Bhandari, Suchismita Acharya

**Affiliations:** 1 Department of Pediatrics, Division of Neonatology, Drexel University School of Medicine, Philadelphia, PA 19102 USA; 2 School of Medicine, Washington University, St. Louis, MO 63110 USA; 3 GenomeRxUS, 1250 Providence Rd, Secane, PA 19018 USA; 4 AyuVis Research Inc, 1120 South Freeway, Fort Worth, TX 76104 USA; 5 Department of Biotechnology, Jamia Millia Islamia, New Delhi, 110025 India; 6 Preclinical Service, University of North Texas Health Science Center, Fort Worth, Texas 76107 USA; 7 University of Cincinnati College of Medicine, Cincinnati, OH 45267 USA; 8 The Warren Alpert Medical School, Brown University, Providence, RI 02903 USA; 9 Acceleration laboratory, University of North Texas Health Science Center, Fort Worth, Texas 76107 USA

## Abstract

In Gram-negative bacterial sepsis, production of excess pro-inflammatory cytokines results in hyperinflammation and tissue injury. Anti-inflammatory cytokines such as IL-10 inhibit inflammation and enhance tissue healing. Here, we report a novel approach to treat septicemia associated with intra-abdominal infection in a murine model by delicately balancing pro- and anti-inflammatory cytokines. A novel oligosaccharide compound **AVR-25** selectively binds to the TLR4 protein (IC_50_ = 0.15 µM) in human peripheral blood monocytes and stimulates IL-10 production. Following the cecal ligation and puncture (CLP) procedure, intravenous dosing of **AVR-25** (10 mg/kg, 6–12 h post-CLP) alone and in combination with antibiotic **imipenem** protected both young adult (10–12 week old) and aged (16–18 month old) mice against polymicrobial infection, organ dysfunction, and death. Proinflammatory cytokines (TNF-α, MIP-1, i-NOS) were decreased significantly and restoration of tissue damage was observed in all organs. A decrease in serum C-reactive protein (CRP) and bacterial colony forming unit (CFU) confirmed improved bacterial clearance. Together, these findings demonstrate the therapeutic ability of **AVR-25** to mitigate the storm of inflammation and minimize tissue injury with high potential for adjunctive therapy in intra-abdominal sepsis.

## Introduction

Sepsis is the leading cause of death in United States hospitals; and elderly and immunocompromised patients are particularly at risk. Sepsis is also among the top conditions for the most expensive hospital stays in the nation and worldwide. More than 900,000 sepsis cases occur each year in the United States, with an annual total cost of approximately $27 billion^1^. To date, three broad approaches to adjunctive (non-antibiotic) therapy have been considered for the treatment of sepsis: (1) optimizing oxygen delivery through oxygenation/ventilation strategies and fluid/vasopressor use to maintain peripheral perfusion, (2) reducing bacterial virulence factors via anti-endotoxin antibodies and endotoxin removal columns and (3) targeting host response factors using corticosteroids, anti-cytokine drugs, and anticoagulants. However, current therapies with these agents are minimally effective in patients with sepsis and their lack of efficacy is more pronounced in immunocompromised and older patients^2^. Hence, there is a huge unmet medical need to develop an effective sepsis therapy.

Inflammatory mediators play a critical role in the pathogenesis and potential management of intra-abdominal sepsis. Animal and clinical data indicate that following bacterial peritonitis, an immense intraperitoneal compartmentalized cytokine response occurs with high levels of certain pro-inflammatory cytokines such as tumor necrosis factor-alpha (TNF-α) and interleukin 6 (IL-6)^3,4^. This cytokine response may be responsible for the uncontrolled activation of the systemic inflammatory cascade.

In sepsis cases caused by Gram-negative bacteria, the bacterial endotoxin lipopolysaccharide (LPS) activates the immune system through signaling via the MD2–toll-like receptor 4 (TLR4) complex to initiate the production of inflammatory cytokines (TNF-α, IL-1β, IL-6) responsible for hyper-inflammation^5,6^. Many researchers are pursuing the development of antagonists that block TLR receptors either by inhibiting activation of TLRs or signaling pathways downstream of TLRs^7^. However, complete inhibition of TLR4 may impose deleterious effect on the immunocompromised state of septic patients who succumb to secondary infection. Eritoran, an LPS analog^8^ failed to improve outcome in a large phase 3 trial and TAK-242, a TLR4 signaling inhibitor, was not pursued after a phase 2 clinical trial. Thus, new therapeutic approaches are needed to effectively inhibit the hyperinflammation produced by the overwhelming cytokine response during bacterial infection without developing resistance to secondary infection in abdominal infections like peritonitis. We approached this issue with a small natural product derived oligosaccharide with TLR4 modulating activity that delicately balances the immune system and the production of anti-inflammatory and pro-inflammatory cytokines in a murine model of peritonitis.

**Figure 1:**
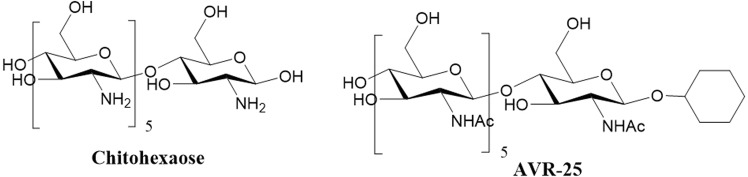
Structure of chitohexaose and **AVR-25**.

High molecular weight (HMW) chitosan is an oligosaccharide with hypocholesterolemic, antimicrobial, immunostimulating, antitumor, anti-inflammatory and antioxidant activities^9^. Chitosan enhances the functions of polymorphonuclear neutrophils, macrophages, and fibroblasts to promote wound healing and can initiate the alternative complement pathway to inhibit sepsis caused by Gram-negative bacteria^8,10–12^. Previous work has shown that, low molecular weight (LMW) chitosan could be a universal antimicrobial agent against Gram-positive and Gram-negative bacteria and fungi^13,14^. We reported that LMW chitosans, such as chitohexaose (Fig. 1) activates macrophages to a non-inflammatory phenotype (alternatively activated macrophages) via a non-canonical TLR4 signaling pathway to produce IL-10^15^. Alternatively activated macrophages have increased phagocytic activity and are involved in tissue repair^16,17^. Chitohexaose blocks the induction of inflammatory mediators induced by LPS, such as TNF-α and IL-6 both *in vitro* (murine macrophages and human monocytes) and *in vivo* (LPS-induced endotoxemia mice models).

Since sepsis usually begins with a local infection in the lungs or peritoneal cavity^18^, murine models of pneumonia and peritonitis have been extensively used to study sepsis. LPS-induced endotoxemia does not represent a clinically equivalent polymicrobial sepsis involving both Gram-positive and Gram-negative bacterial infections. The discrepancy in LPS sensitivity between mice and humans, where mice are resistant to the toxic effects of bacterial LPS^19,20^ and humans are exquisitely sensitive, also suggests that data obtained using murine models of LPS-induced sepsis may not apply to human illness. Cecal ligation and puncture (CLP), a murine model of bacterial peritonitis, is regarded as the clinically equivalent animal model of sepsis. CLP in mice recapitulates key features of secondary bacterial peritonitis in humans, including polymicrobial infection^21^, persistently elevated circulating high mobility group box 1 (HMGB1) protein levels^22^, hyperdynamic circulatory system^23^, and the development of acute lung injury^24^. We have undertaken a rational and robust drug discovery and development approach to find an optimized compound with a suitable preclinical profile, potency and efficacy in the CLP-induced polymicrobial sepsis model.

In this study we have investigated a new treatment option for sepsis based on the following rationale: (1) Chitohexaose is a highly positively charged molecule, and has fast clearance and a short half-life^25^ in plasma; hence, structural optimization of these class of compound is necessary to improve their bioavailability. (2) A study with multiple time points is needed to understand the dynamic changes of cytokine levels, and a pharmacokinetic study of the compound is required to determine the necessary dosing frequency. (3) As sepsis affects all ages and both sexes, the efficacy of the compound needs to be examined in both male and female young adult and aged mice.

## Results

### Structural modification of Chitohexaose

Our modified compound **AVR-25** (N-((2R,3R,4R,5S,6R)-5-(((2S,3R,4R,5S,6R)-3-acetamido-4,5-dihydroxy-6-(hydroxymethyl)tetrahydro-2H-pyran-2-yl)oxy)-2-(cyclohexyloxy)-4-hydroxy-6-(hydroxymethyl)tetrahydro-2H-pyran-3-yl)acetamide) was selected as the lead analog (Fig. 1) to study this disease. *In silico* modelling demonstrated that **AVR-25** has a unique binding mode similar to N-hexaacetyl chitohexaose, in which it either binds to the dimerization site or to the middle region of the TLR4 protein but not to the LPS binding site^15^. This unique binding mode possibly restricts the dimerization of TLR4 and interrupts the downstream signaling cascade.

### Safety profiling of AVR-25

The initial mutagenicity and hERG (human ether-à-go-go-related gene) channel activity were assessed *in vitro* for compound **AVR-25** as part of non-clinical safety profiling using a modified Ames test. The objective of the Ames bacterial mutation assay was to determine whether compound **AVR-25**, at different concentrations, induces reverse mutations in histidine-requiring bacterial strain of *Salmonella typhimurium* (TA-100), both in the absence and presence of a rat liver metabolizing system. When the bacterial strains are exposed to a mutagen, some of the bacteria in the treated population undergo genetic changes that cause them to revert to a prototrophic state and thus grow in the absence of exogenous amino acids. In our profiling study, there was no evidence of toxicity or precipitation in strain TA-100 in the absence or presence of human liver S-9 treated with **AVR-25** (Supplementary Table S1). Further, there was no notable increase in revertant numbers observed in strain TA100 tested both in the absence and presence of S-9 that would be considered an evidence of mutagenic activity. The *in vitro* effects of **AVR-25** on the hERG potassium channel current (a surrogate for IKr, delayed rectifier cardiac potassium current) expressed in mammalian cells were evaluated (in triplicates) at room temperature for 5 min at concentrations of 0.3, 3, 30 and 300 μM. **AVR-25** did not inhibit the hERG channel and IC_50_ was >300 µM, thus confirming no adverse cardiac effects (Supplementary Table S2).

### AVR-25 upregulates the anti-inflammatory and down regulates the proinflammatory cytokines via binding to TLR4 in human peripheral blood monocytes (hPBMC)

**Figure 2:**
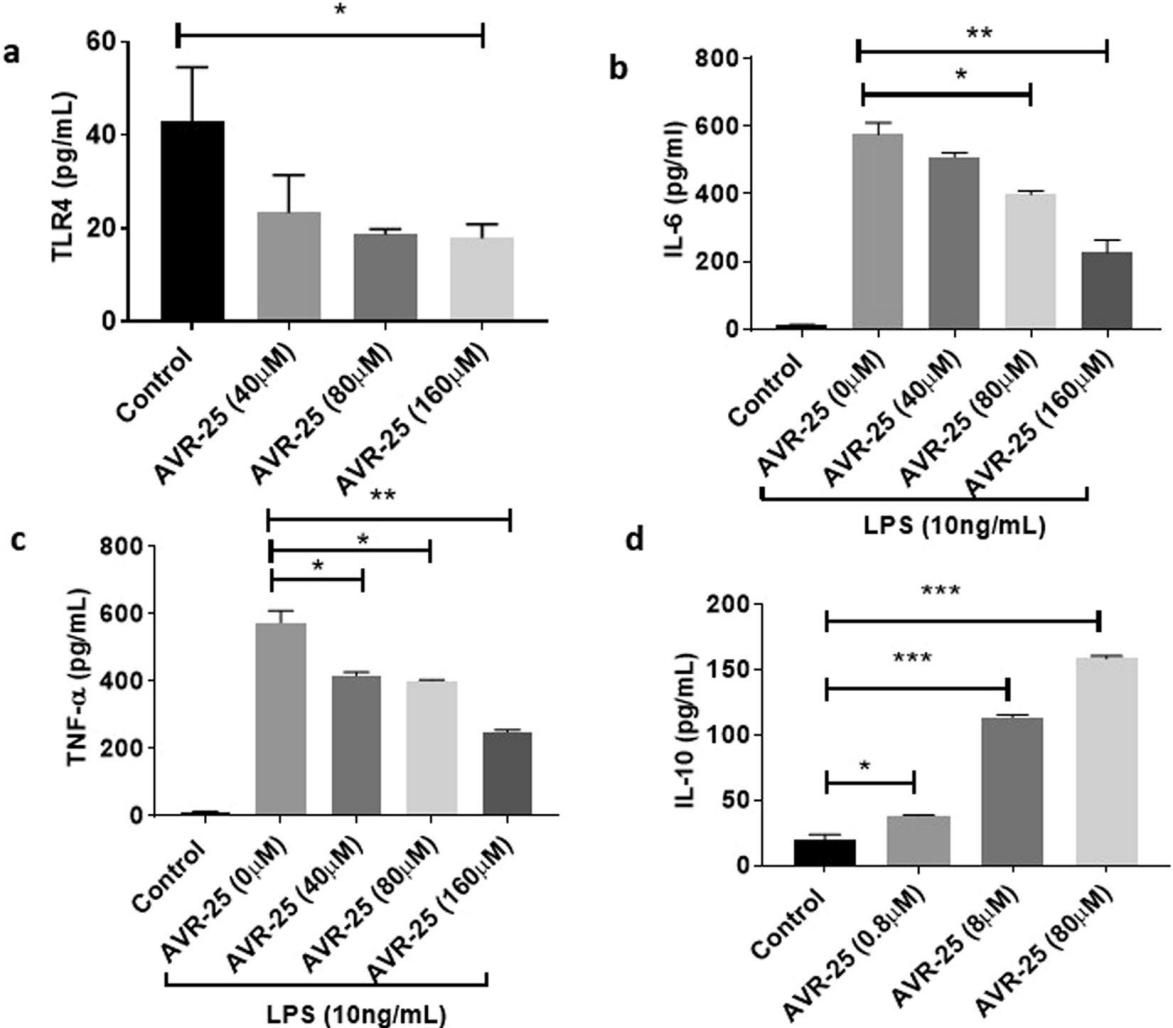
Effect of **AVR-25** in human PBMCs as shown by ELISA assay with or without LPS treatment. (**a**) Binding of **AVR-25** to TLR4 at different concentrations. (**b**) **AVR-25** inhibited LPS (100 ng/mL) induced IL-6 and (**c**) TNF-α. (**d**) **AVR-25** induced IL-10 production in PMBCs. Control indicates untreated cells in media. N = 4; *p < 0.05, **p < 0.005, ***p < 0.0001.

As previously reported by Panda *et al*.^15^, in a mouse model of endotoxemia, the storm of inflammation induced by LPS is delicately balanced by LMW chitohexaose through TLR4, by activating the macrophages into a non-inflammatory phenotype (alternate activation). To test the immunomodulatory property of **AVR-25**, we added different concentrations of **AVR-25** (0.8, 8.0 and 80 µM) to hPBMCs (LeukoPak) with or without LPS (100 ng/mL) for 48 h. The supernatant was used to assay IL-10, TNF-α, and IL-6 levels and the cell lysate for TLR4 and TLR2, by ELISA. We found that the alternate activation of macrophages by **AVR-25** regulated the modulation of both pro- and anti-inflammatory cytokines at concentrations of 8–80 μM (Fig. 2a–d). At 80 μM, the binding of **AVR-25** was maximum with TLR4 (Fig. 2a). Treatment of LPS followed by treatment with **AVR-25** at increasing concentrations decreased the expression of the pro-inflammatory cytokines IL-6 (Fig. 2b) and TNF-α (Fig. 2c) at 160 μM. Treatment alone with **AVR-25** at a concentration of 8 µM increased the expression of the anti-inflammatory cytokine, IL-10 (Fig. 2d). This demonstrates that, **AVR-25** binds to TLR4 and inhibits LPS-induced proinflammatory cytokine production in a dose-dependent manner. The IC_50_ of **AVR-25** was determined to be 0.15 µM for TLR4 binding after a dose response study (Supplementary Fig. S1). Compound **AVR-25** did not have any binding affinity for TLR2 at 10 µM (Supplementary Fig. S2).

### AVR-25 improves survival in both young adult and aged mice from CLP-induced polymicrobial sepsis and death

**Figure 3:**
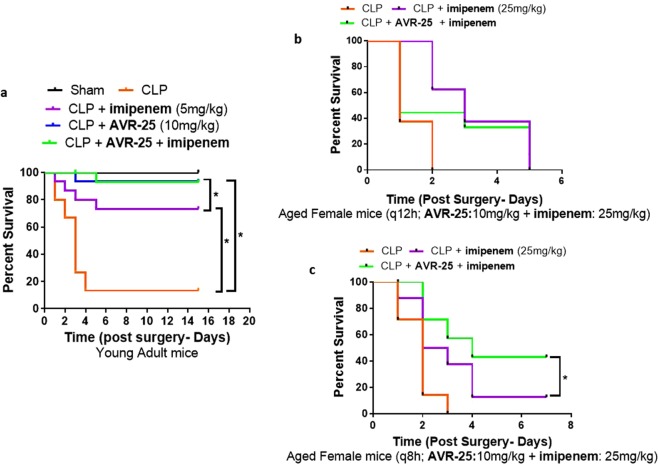
**AVR-25** increases survival in both young adult and aged mice. **(a)** Two doses of **AVR-25** (10 mg/kg; 16 h, 24 h) were injected IV, into 10–12-week-old young adult mice alone, or in combination with **imipenem** (5 mg/kg), and survival was followed up to 15 days. Following CLP, the **AVR-25** + **imipenem** group demonstrated better survival (>80%) than the group treated with **imipenem** alone or **AVR-25** alone. There was no mortality in the sham group, while in the CLP group there was >90% mortality within 72 h of the procedure, N = 5–15, *p < 0.05. **(b) AVR-25** (10 mg/kg) was injected into aged female mice (16–18 months old) 6 h post CLP and q12h followed by **imipenem** injection SC (25 mg/kg) every 12 h for 7 consecutive days. N = 8–10; *p < 0.05. Sham-Black; CLP-orange; CLP + **imipenem**: Purple; CLP + **AVR-25**: Blue; CLP + **AVR-25** + **imipenem**: Green. Sham groups and **AVR-25** alone were not done for (**b** and **c**). (**c**) **AVR-25** (10 mg/kg) was injected into aged female mice (16–18 months old) 6 h post CLP, q8h followed by **imipenem** injection SC (25 mg/kg) every 12 h for 7 consecutive days. In the aged group, **AVR-25** + **imipenem** demonstrated improved survival (42%) as compared to **imipenem** alone (~20%). Administration of **AVR-25** alone was not done in this group. N = 8–10.

**AVR-25** has a binding potency similar to the binding of chitohexaose with TLR4 (Supplementary Fig. S2); hence, we hypothesized that, compound **AVR-25** will have similar therapeutic efficacy as chitohexaose in intra-abdominal infection modeled by CLP mice, either alone, or in combination with standard of care antibiotics. To test this, we injected 2 doses of **AVR-25** (10 mg/kg, intravenous (IV), 16 h and 24 h post CLP) in young (10–12 weeks old, male and female) mice. The dose was selected based on our earlier study where 10 mg/kg of chitohexaose, when dosed intraperitonially, was effective in protecting mice against *E. coli*-induced septicemia (unpublished). In the current study, **AVR-25** protected mice against CLP-mediated organ injury and death, either alone or in combination with the standard point of care antibiotic **imipenem** (imipenem was used in combination with cilastatin (Primaxin®; Carbosynth Inc.), hereafter referred to as **imipenem** only; 5 mg/kg, subcutaneous; SC)^26^. Survival was monitored for 15 days. All the mice in the CLP group died within 72 h post-surgery while the groups treated with **imipenem** alone or **AVR-25** alone or with a combination of **AVR-25** + **imipenem**, survived for up to 2 weeks (Fig. 3a). In all the above 3 groups, there was >75% survival, and females responded better than males (not shown). Although, there was no significant difference in survival between **imipenem**, **AVR-25** and **AVR-25** + **imipenem** treated groups, the group treated with **imipenem** alone, were unable to recover from the septic shock/syndrome completely. They appeared sick, did not feed properly and displayed abnormal behavioral parameters. On the other hand, mice receiving a combination of **AVR-25** + **imipenem** had comparable physical appearance, feeding and behavioral parameters to normal control (non-operated) mice. Hence, translationally, combining **AVR-25** to **imipenem** improved the overall health of mice, in addition to just survival alone (Fig. 3a**)**.

At the same time, we also assessed the effectiveness of **AVR-25** in improving the survival of aged mice (16–18 months old, human equivalent to 50–70 years old^27^) to mimic the clinically equivalent aged population under standard primary care (fluid resuscitation, antibiotic therapy) that succumb to sepsis. To recapitulate the clinical situation where there is a delay between the insult (CLP) and the onset of therapy, antibiotic therapy was initiated 30 min post CLP and repeated every 12 h for 7 days post-surgery along with saline. A clinically relevant antibiotic regimen consisting of 25 mg/kg of **imipenem** delivered SC, was used for aged mice, providing coverage against both aerobic and anaerobic bacteria^28^.

Both the aged male and female mice were administered 10 mg/kg **AVR-25** IV, at 6 h post CLP, then q12h (twice a day, Fig. 3b) and q8h (three times a day, Fig. 3c) for 7 days post-surgery along with 25 mg/kg **imipenem** (q12h) in two separate studies. This regimen was adopted to mimic the geriatric population, clinically^28^. **AVR-25** dosed q8h (Fig. 3c) in combination with **imipenem** was more effective (42%) in protecting the female aged mice from death than those dosed at q12h (22%, Fig. 3b). Males responded poorly to q12h doses (12% survival, data not shown), suggesting the need for further dose optimization.

### AVR-25 restores organ damage following polymicrobial infection

Antibiotics alone are not always effective in treating the multifaceted pathological complexity of intra-abdominal sepsis (infection, inflammation, immune suppression). Sepsis develops when the inflammatory response to infection rises to a critical level and physiologic alterations occur when there is a burst of cytokines. The high bacterial load or virulence can cause an exaggerated inflammatory response, resulting in tissue damage and organ dysfunction. To test whether **AVR-25** restored organ damage against sepsis-induced injury, we harvested tissues from vital organs that easily succumb to infection such as the lungs, kidney, liver, heart, spleen, brain, gut, and testes/ovaries, from all experimental groups to study histopathological changes. All the tissues from different experimental groups were harvested after 72 h. This is because the CLP only group did not survive beyond 72 h while the other treated groups (**imipenem** only, **AVR-25** only, **AVR-25** + **imipenem**) lived longer. Scoring was done following the methodology adopted from Kubiak *et al*.^29^ and Klopfleisch^30^. The criteria of scoring used in this study is summarized in Supplementary Table S3 and highlighted in Supplementary Fig. S3.

**Figure 4:**
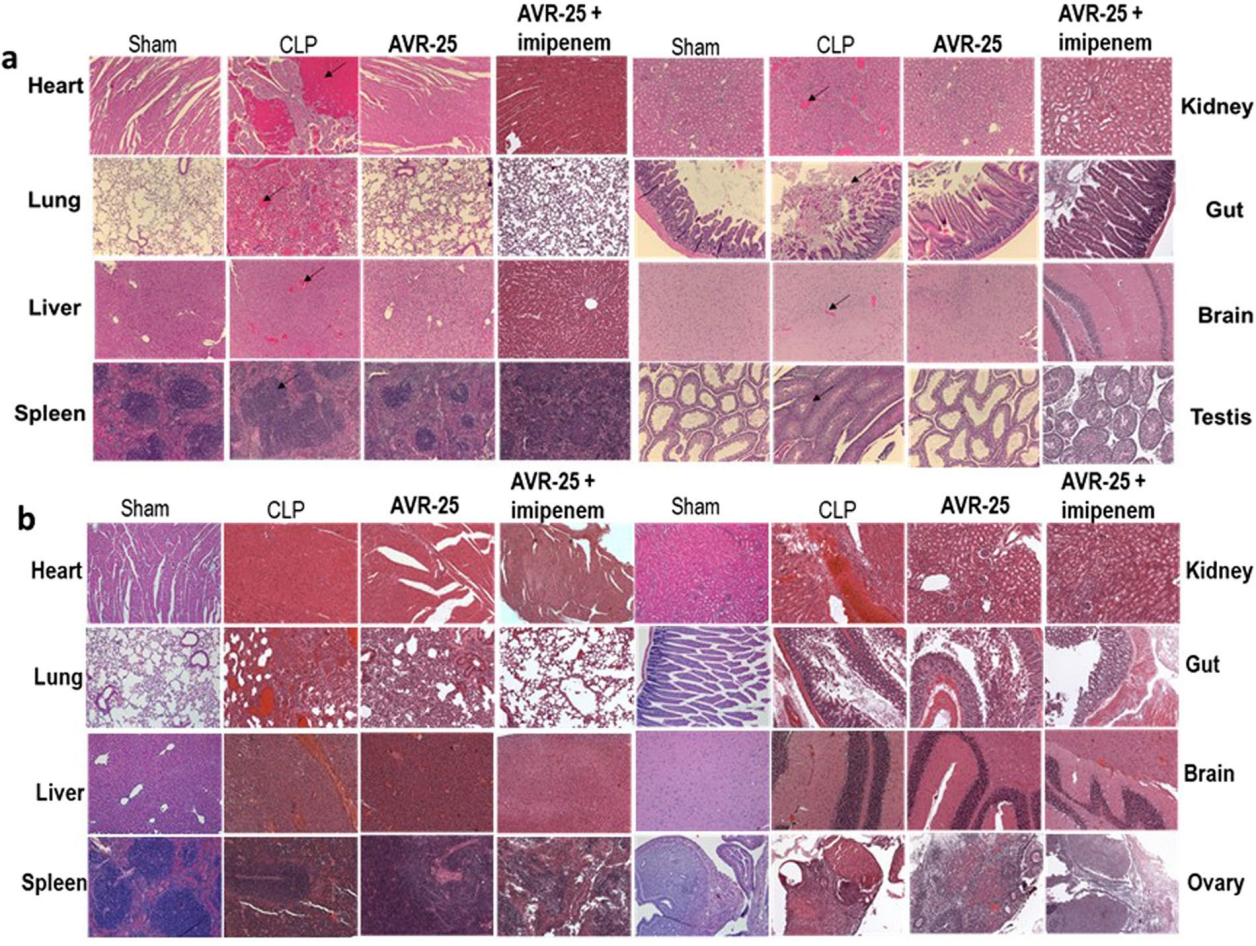
**AVR-25** restores normal organ pathology. Representative H/E staining showing restoration of normal tissue morphology in vital organs of both male **(a)** and female **(b)** young adult mice in **AVR-25** + **imipenem** treated mice compared to the CLP group. CLP mice showed microthrombi and congestion (arrows) in the heart, lungs, liver, kidney, and brain, increased germinal center size in spleen, necrosis of villi in the gut and loss of testicular and ovarian epithelium. Upon treatment with **AVR-25** + **imipenem**, the histology showed a marked improvement in controlling the damage and the vascular changes. These changes appeared to be superior to those of the **imipenem** only treated group, N = 15, **X100**.

**Figure 5:**
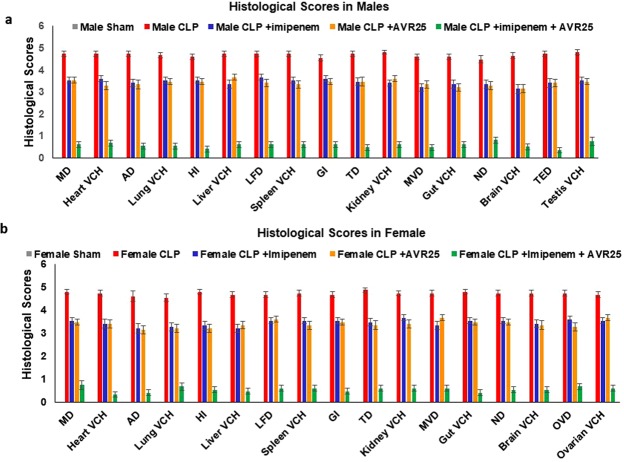
Compound **AVR-25** recovered CLP induced tissue damage. The graph shows histological scoring (summarized in supplemental section, Table S3) in CLP-induced **(a)** male and **(b)** female mice. The recovery of all tissue damage was maximum when treated with **AVR-25** + **imipenem** than with **AVR-25** alone or **imipenem** alone. Vascular damage and tissue congestion induced by CLP in heart, lungs, liver, spleen, kidney, gut, brain, testis and ovary is restored after drug treatment. In all the tissues studied P < 0.01 (CLP vs **imipenem** alone, **AVR-25** alone); P < 0.001 (**imipenem** alone or **AVR-25** alone vs **AVR-25** + **imipenem** in both males and females. Higher scores indicate more injury. In both (**a** and **b**), Sham is indicated by grey color; CLP-Red; CLP + **imipenem** alone-Blue; CLP + **AVR-25** alone-Orange; CLP + **AVR-25** + **imipenem** -Green. As the average score for the sham group is 0, the grey color is not visible in the bar graphs. **MD**: Myocardial Damage; **Heart VCH**: Heart Vascular Congestion & Hemorrhage; **AD**: Alveolar Damage; **Lung VCH**: Lung Vascular Congestion & Hemorrhage; **HI**: Hepatocyte Injury; **Liver VCH**: Liver Vascular Congestion & Hemorrhage; **LFD**: Lymphoid Follicles Damage; **Spleen VCH**: Spleen Vascular Congestion & Hemorrhage; **GI**: Glomeruli Injury; **TD**: Tubular Damage (kidney); **Kidney VCH**: Kidney Vascular Congestion & Hemorrhage; **MVD**: Mucosal Villi Damage; **Gut VCH**: Gut Vascular Congestion & Hemorrhage; **ND**: Neuronal Damage; **Brain VCH**: Brain Vascular Congestion & Hemorrhage; **TED**: Tubular Epithelial Damage; **Testis VCH**: Testis Vascular Congestion & Hemorrhage; **OVD**: Ovarian Follicular damage; **Ovarian VCH**: Ovary Vascular Congestion & Hemorrhage.

Tissues from the CLP group showed microthrombi and congestion in the heart, lungs, liver, kidney and brain, increased germinal center size in spleen, necrosis of villi in gut, and loss of testicular epithelium; Tissue damage was more prominent in the **imipenem** alone treated group (Supplementary Fig. S3); treatment with **AVR-25** alone reversed these changes in all organs studied, in both males and females (Supplementary Fig. S4); hemorrhage was decreased, though not completely abolished. However, treatment with **AVR-25** + **imipenem** reversed the changes towards a normal phenotype so as to resemble the sham group; there was no trace of hemorrhage (Fig. 4). Figure 5a,b show the scoring summary of tissue damage after CLP and recovery following treatment with **AVR-25** either alone or in combination with **imipenem**, in both male Fig. 5a) and female (Fig. 5b) young adult mice. Similar changes were also observed in aged mice following CLP (Supplementary Fig. S4)

### AVR-25 modulates the immune response *in vivo*

**Figure 6:**
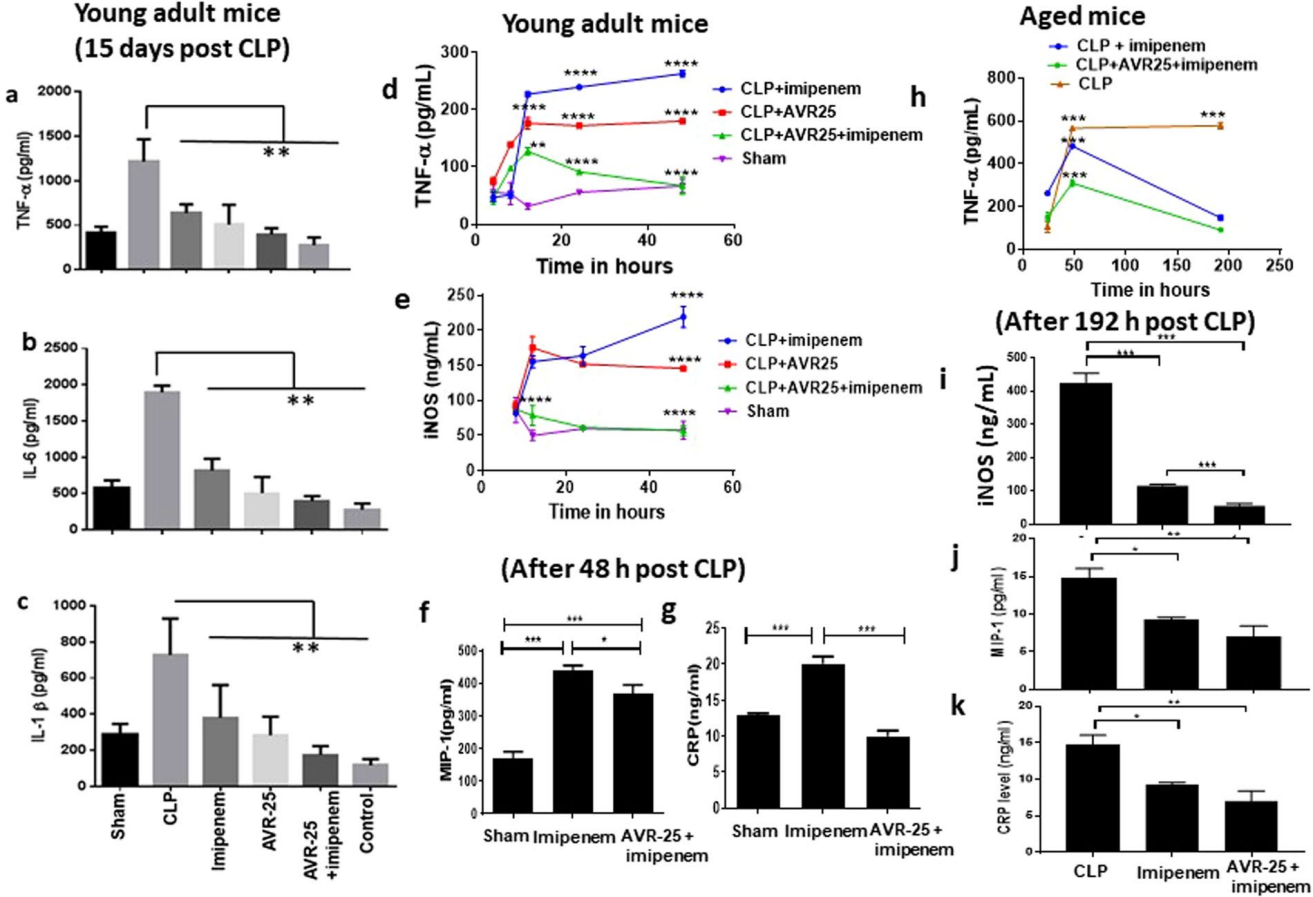
Changes in pro-inflammatory markers with AVR-25 treatment. (**a**–**c**) Pro-inflammatory cytokines in serum after 15 days in young adult mice (male and female, N = 10). (**d**,**e**) Dynamic changes in pro-inflammatory cytokines after 4, 8, 12, 24, and 48 h of treatment with **AVR-25** + **imipenem** in CLP young adult mice. (**f**,**g**) CRP and MIP-1 levels measured after 48 h. (**h**) Dynamic changes in pro-inflammatory cytokines after 24, 48, 72, and 192 h of **AVR-25** and **imipenem** treatment in aged CLP mice. i-k) i-NOS, CRP, and MIP-1 levels measured after 192 h. *p < 0.005 for (**a**–**c**). *p < 0.01, **p < 0.001, ****p < 0.0001 for Figs d–k were found between the CLP + **imipenem** and CLP + **AVR-25** + **imipenem** groups as well as between sham and CLP + **AVR-25**. There was no difference between results obtained from male and female mice.

Following infection, cytokines, such as IL-1β, IL-6, TNF-α, and inducible nitric oxide synthetase (i-NOS), that coordinate immune/inflammatory responses are triggered and activated. We wanted to assess the immunomodulatory property of **AVR-25** in the CLP mouse model by studying the change in expression of several pro- and anti-inflammatory cytokines at different time points. We conducted two independent studies in two different laboratories and collected serum from all experimental groups to perform ELISA on a panel of pro- and anti-inflammatory cytokines. Our first study from one laboratory (Fig. 6a–c), demonstrated a significant decrease (p < 0.001) in serum pro-inflammatory cytokines TNF-α, IL-1β, and IL-6 after 15-days post CLP treatment with **AVR-25** + **imipenem** in young adult mice (10 mg/kg, two doses at 16 h, 24 h post CLP). The second experiment from a separate laboratory demonstrated time-dependent changes (Fig. 6d–g) of pro-inflammatory cytokines TNF-α and i-NOS at 4, 8, 12, 24, and 48 h after administration of **AVR-25** + **imipenem** to CLP induced young adult mice (10 mg/kg, 6 h+, q8d). Macrophage inflammatory protein-1 (MIP-1) and C-reactive protein (CRP) levels were decreased in **AVR-25** + **imipenem** treated group after 48 h. Due to technical limitation, we could not collect samples at earlier timepoints for these assays. As expected, the key inflammatory biomarker TNF-α, was upregulated as early as 8 h post CLP in the **imipenem** only treated group and peaked at 48 h (Fig. 6d). The level of TNF-α was significantly lower (p < 0.05) in the **AVR-25** + **imipenem** group as compared to the **imipenem** only group. Levels of i-NOS, MIP-1 and CRP were also lower in **AVR-25** + **imipenem** group when compared to **imipenem** group only. A similar trend in change in cytokine levels was observed in aged mice (16–18 months old) at 24, 48, 72, and 192 h after administration of **AVR-25** to CLP mice (10 mg/kg, 6 h+, q8d) as shown in Fig. 6h–k.

**Figure 7:**
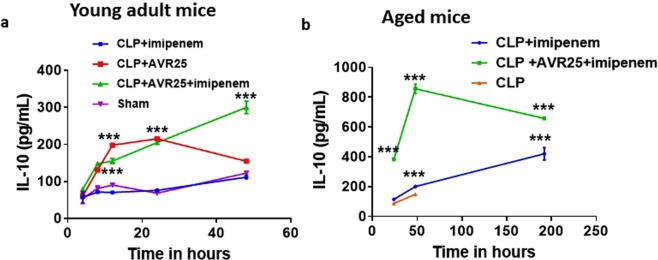
(**a**) Change in anti-inflammatory cytokine IL-10 in serum of young adult mice (male and female, N = 10) after 4, 8, 12, 24, and 48 h of treatment with **AVR-25** + **imipenem** post CLP. (**b**) Change in IL-10 in serum of aged mice (male and female, N = 8) after 24, 48, 72, and 192 h of treatment with **AVR-25** + **imipenem** post CLP. ***p < 0.0001 was found between CLP + **imipenem** and CLP + **AVR-25** + **imipenem** groups as well as between sham and CLP + **AVR-25**. There was no difference between results obtained from male and female mice.

IL-10 is known to be responsible for tissue protection, possibly by decreasing the overproduction of pro-inflammatory cytokine TNF-α^31^. Here, we measured IL-10 levels in plasma serum. As expected, we observed a statistically significant increase (p < 0.0001) in the anti-inflammatory cytokine IL-10 in both the **AVR-25** and **AVR-25** + **imipenem** treated group between 12–48 h (Fig. 7a) in young adult mice, and between 24–192 h in aged mice (Fig. 7b) both dosed 10 mg/kg, 6 h+, q8d. There was increased expression of IL-10 in the serum, along with restoration of organ morphology and histology of all tissues in the **AVR-25** + **imipenem** treated group.

## Discussion

Sepsis continues to be the major cause of death in intensive care units. Despite intensive research worldwide, a new drug that effectively reduces mortality due to sepsis is still needed. An LMW naturally derived oligosaccharide analog **AVR-25** has proved effective in combating this dreadful condition, as is evident from the findings of our present study in a mouse model of CLP-induced sepsis. In anticipation to improve the plasma bioavailability of **AVR-25**, we derivatized all six amino groups of chitohexaose to hexaacetate and substituted the anomeric hydroxyl group with a cyclohexyl group to generate the N-hexaacetylated chitohexaose analog compound **AVR-25**.

Many previous studies have demonstrated the role of TLR2 and TLR4 inhibition in the pathogenesis of sepsis. The hallmark of sepsis-induced immune dysfunction includes depletion of dendritic cells in secondary lymphoid organs. Sepsis-induced acute kidney injury^32^ in C57BL/6 TLR2^−/−^, TLR4^−/−^ and MyD88^−/−^ male mice subjected to CLP had lower neutrophil infiltration in the kidneys, leading to less inflammation and better renal protection. Apoptosis of spleen dendritic cells was reportedly increased in septic wild-type mice and inhibited in TLR4 knockout mice^33^. Hence, modulation of TLR4/TLR2 via competitive inhibition of corresponding ligand (LPS or zymosan) binding or modulation of the receptors to produce non-inflammatory cytokine phenotypes may decrease the hyper inflammatory response and maintain the dendritic and T cell populations needed to preserve innate immunity.

In rats (9–10 weeks old) challenged with *Escherichia coli*, Eritoran demonstrated maximum effectiveness when dosed 1 h before challenge^34^; however, the efficacy diminished when administered at 1 h and 3 h (2 doses) after the challenge. Similarly, another potent TLR4 signal transduction inhibitor TAK242 increased the survival of mice (8–9 weeks old) from 17% to 51% when dosed 1h-4h after CLP-induced sepsis^35^. Ironically, both drugs failed to treat sepsis patients in clinical studies. In this study, we have demonstrated that **AVR-25** effectively protected mice from CLP-induced death and organ injury when dosed >6 h post CLP, perhaps providing a larger window for therapy.

**AVR-25** binds to TLR4 with an IC_50_ of 0.15 µM and is selective for TLR4 over TLR2 protein. Binding to TLR4 activated M2 macrophages and dose dependently increased IL-10 levels in monocytes. IL-10 is a compensatory cytokine known to reduce the level of pro-inflammatory cytokines responsible for tissue injury. Schneider *et al*.^36^ reported that, soon after trauma or hemorrhagic shock, IL-10 regulated the pro-inflammatory cytokine activity via an autocrine effect on cytokine mRNA transcription in Kupffer cells. Kato *et al*.^31^ demonstrated that lethality in septic mice was significantly decreased, and elevated circulating TNF-α was suppressed when 1 µg or more of recombinant murine IL-10 was administered 6 h after induction of sepsis. In both IL-10-deficient and wild-type mice, 5 h post CLP treatment with recombinant human IL-10, significantly improved survival and lengthened the therapeutic window for rescue surgery. Similarly, T van der Poll *et al*.^37^ reported that IL-10 production during endotoxic shock is part of a protective mechanism that involves IL-10-induced inhibition of TNF synthesis, and, in septic peritonitis induced by CLP in mice, there is a rapid induction of IL-10 mRNA. These cumulative results demonstrate that IL-10 controls the onset of irreversible septic shock after CLP and that, a similar mechanism may also apply to **AVR-25** for inducing IL-10 in our CLP model, following treatment.

We have previously reported that chitohexaose produced IL-10 in both human monocytes and mouse bone marrow derived macrophages and protected mice against LPS-induced endotoxemia. In our current CLP mouse model of abdominal sepsis, both early (6 h post CLP) and late (12 h post CLP), intervention of 10 mg/kg of **AVR-25** significantly reduced the pro-inflammatory cytokines as well as CRP^38^.

Based on the effective dose for chitohexaose in the mouse endotoxemia model^15^, we selected 10 mg/kg as our IV dose for young adult (10–12 weeks old) mice dosed 16 h after CLP. Two doses (16, 24 h) of compound **AVR-25** significantly increased survival of CLP mice from 11% to 93% and minimized histopathological damage in all organs 15 days post dose. The serum level of pro-inflammatory cytokines (TNF-α, IL-1β and IL-6) showed a decreasing trend and was lowest in the **AVR-25** + **imipenem** treated group when compared to CLP group. Further, the **imipenem** only treated group had higher cytokine levels when compared to the **AVR-25** only and **AVR-25** + **imipenem** groups. Thus, we only compared the dynamic change of pro-inflammatory (TNF-α, i-NOS) and anti-inflammatory markers (IL-10) between **imipenem** only, **AVR-25** only and **AVR-25** + **imipenem** groups. As expected, there was a time-dependent increase in the levels of TNF-α and i-NOS in the **imipenem** alone treated group, which was significantly decreased in the **AVR-25** alone or in **AVR-25** + **imipenem** group, indicating the existence of a synergistic therapeutic effect of **AVR-25** and existing beta-lactam standard of care antibiotic **imipenem**.

CRP is considered to be a marker of bacterial load^38^; CRP levels were significantly decreased at 48 h and 192 h in the **AVR-25** + **imipenem** treated group, indicating effective bacterial clearance. Increased IL-10 levels in the **AVR-25** + **imipenem** group correlated with the reversion of histopathological changes to normal, supporting our hypothesis that, enhanced production of IL-10 not only neutralizes the cytokine storm but also protects the tissues against excessive damage. We did not find any sex-specific difference in the survival or in the cytokine levels between 10–12 week-old male and female mice, despite earlier reports documenting that there is a sex-specific immune response in sepsis and that male mice responds poorly to immunotherapy^39^. From our experiments, we confirmed that in the **AVR-25** only treated group, female mice had better survival (90%) as compared to male mice (75%, data not shown). Female aged mice IV administered 10 mg/kg **AVR-25** dosed q8h in combination with **imipenem** had improved survival (42%) when compared to those dosed q12h (22%). This result indicates an opportunity for further dose frequency optimization. The serum data demonstrates a decreasing trend in total serum bacterial CFU/mL after 48 h of treatment (Supplementary Fig. S5). There was no improvement in the survival of aged male mice (~15%) even with a combination of **AVR-25** + **imipenem** dosed q12h. When the dose of **AVR-25** was escalated to 20 mg/kg (q12h), there was a trend in increase in survival (data not shown), indicating an existing opportunity to improve the survival by optimizing the amount and frequency of the dose.

The key findings from this study were that aged mice are highly susceptible to sepsis-induced death with increased pro-inflammatory cytokine levels in serum and profound tissue damage. **Imipenem** alone could not protect tissues while **AVR-25** alone resulted in decreased tissue damage, though not in complete recovery. A combination of **AVR-25** + **imipenem** improved survival with minimum tissue injury and restoration of behavioral parameters. We confirmed our result in aged mice with previous reports^28^ to show that there is a sex-dependent difference in response to sepsis and recovery and male mice are more vulnerable than female mice. Dose titration of **AVR-25** in aged mice may result in better survival, with further dose optimization. Additionally, **AVR-25** demonstrated an acceptable safety profile as determined by hERG channel, Ames, and *in vitro* micronucleus assays.

Pro-inflammatory cytokines are critical in eliminating infection; yet excessive production can cause tissue and organ damage. The unique feature of **AVR-25** includes the ability to delicately balance the pro- and anti-inflammatory cytokine level via upregulating IL-10 while simultaneously downregulating TNF-α, IL-1β, i-NOS, and CRP. Here, we report the therapeutic potential of a naturally derived oligosaccharide analog with the ability to increase survival and minimize tissue damage in mice with intra-abdominal sepsis. Our results clearly demonstrate that **AVR-25** alone is highly effective (>80%) in protecting young adult mice (10–12 weeks old, human equivalent to 25–35 years old^27^) from sepsis-induced mortality. Additionally, compound **AVR-25** in combination with **imipenem** decreased mortality (>40%) and protected against organ dysfunction in aged (16–18 months old, human equivalent to 50–70 years old^27^) mice, where antibiotic treatment alone was ineffective.

## Methods

### Animals

Two different age groups of wild-type C57BL/6 mice (young adult: 10–12 weeks old, aged: 16–18 months old) from both sexes were obtained from Jackson Laboratories (Bar Harbor, Maine Harbor, ME, USA). The CLP procedure was performed in accordance with the NIH Guide for the Care and Use of Laboratory Animals and methods were approved by the Institutional Animal Care and Use Committee (IACUC) of University of North Texas Health Sciences Center, Texas and Drexel University, Philadelphia.

### Cecal Ligation and Puncture (CLP) study

CLP was done following the method adopted by Rittirsh *et al*.^40^. A total of 90 young adult mice (10–12 weeks old), and 100 aged mice (16–18 months) were used for the studies. For the survival studies, histopathology and biomarker analysis, we used 10–15 mice for each group. For time dependent biomarker analysis study, we used young adult mice (N = 5) and aged mice (N = 8–15 males and females) per group.

Prior to the procedure, abdominal hair was removed from the animals using commercially available Johnson’s hair remover cream. The animals were anesthetized with Xylazine-Ketamine (10 mg/ml/kg body weight). Then, a 1-cm incision was made gently within the disinfected area from a cranial to caudal aspect, parallel to the midline near the linea alba. The incision was made in the region where the cecum is located for minimal invasion, and the cecum was gently pulled out of the abdominal cavity. A ligature was made with 2–0 silk suture material around the cecum’s external wall, just beneath the ileocecal junction. The ligated cecum was next perforated (mesenteric to anti-mesenteric) with a 25 G needle (BD Biosciences) and the cecal contents were exposed through the perforation. The cecum was placed back into the peritoneal cavity and the incision through the peritoneal wall was stitched and closed. Sterility was maintained throughout the procedure by operating the animals under a laminar hood. Following the procedure, the animals were returned back to the animal facility for subsequent monitoring and follow up. Body temperature was recorded every 24 h, and feeding behavior was observed. Mice were fed semisolid food for 48 h before resuming their regular chow diet. The sham mice had the abdominal cavity opened, the cecum was not perforated, and the peritoneum was stitched back. CLP animals underwent the procedure, as described above. In the **imipenem** only group, after the CLP procedure, **imipenem** was given subcutaneously at 5 mg/kg 30 min after the procedure (1 dose) to 10–12-week-old mice and 25 mg/kg to 16–18-month-old mice every 12 h for 7 days^28^. In the **AVR-25** + **imipenem** group, **imipenem** was given subcutaneously at 5 mg/kg 30 min after the procedure (1 dose), while **AVR-25** was injected intravenously (in the tail vein) at a dose of 10 mg/kg, in one study with two doses, 16 and 24 h time points and in the second study dosed at every 8 hours for 3 days post CLP in 12-week-old mice. Aged mice (16–18 months old) were IV administered 10 mg/kg **AVR-25** at +6 hrs, then q8h (3 times a day) for 7 days post-surgery along with 25 mg/kg of **imipenem** (q12h). Immediately after surgery, animals were monitored for 2 consecutive weeks for survival, daily activity, and overall behavioral changes, after which they were euthanized, their tissues collected for histopathology, and blood collected (serum) for inflammatory biomarker analysis.

### Histopathology

Whole organs including heart, lung, liver, spleen, kidney, gut, brain, testis, and ovary were collected from different groups of mice and combined into multi-tissue blocks. The tissues were fixed immediately in 10% buffered formalin overnight at 4 °C. Then, tissues were washed, dehydrated in a graded series of ethanol, embedded in paraffin, and sectioned at 5 µm to be stained with Hematoxylin-Eosin. All images were photographed under 10X magnification for overall changes and under 40X magnification to highlight specific changes after damage and following treatment, using an Olympus (DP73) microscope. Scoring was done following the methodology adapted from Kubiak *et al*.^29^ and Klopfleisch^30^ by a board-certified pathologist. Every individual tissue of every single mice from different study groups were assessed for scoring. The following cells in each of the organs was scored on the total percentage of area damage - Heart (Myocardium), Lung (alveoli), Kidney (glomeruli, tubules), Spleen (lymphoid follicles), Liver (Hepatocyte), Brain (Neuronal Damage), Testis (Seminiferous tubules) and Ovary (Follicles). Vascular congestion and hemorrhage were assessed by the number of areas/High power field (HPF).

### Chemicals and reagents

The synthesis and structural characterization of **AVR-25** was performed in the AyuVis Research Laboratory following published protocols^41,42^. N-hexacetyl chitohexaose and chitohexaose were purchased from Carbosynth Inc. Imipenem used in combination with cilastatin (Primaxin®) was purchased from Carbosynth Inc. (San Diego, CA). LPS and phorbol myristyl acetate (PMA) were purchased from Sigma-Aldrich Inc.

### Formulations

The compound **AVR-25** (10 mg/kg) was reconstituted in 0.9% normal saline, and 125 µl was administered intravenously (IV) via the lateral tail vein. **Imipenem** + cilastatin (Primaxin®) was formulated in 0.9% saline solution and administered subcutaneously (SC) at a dose of 5 mg/kg to young adult mice and 25 mg/kg to aged mice in a 0.2 mL volume near the dorsal scapular region.

### Cell lines

Human peripheral blood mononuclear cells (HPBMCs) were purchased from LeukoPaK (AllCells); 0.5 × 10^6^ cells/well were seeded in 96-well plates and allowed to grow at 37 °C for 8–10 h for confluency. THP-1 monocytes were purchased from ATCC and grown to confluency using RPMI-1640 medium with 10% FBS and 1% of Penicillin and Streptomycin. 1 × 10^5^ cells/well were seeded in 96-well plates and stimulated with 200 nm of PMA (Phorbol-12-myristate-13-acetate) for 48 h to induce macrophage differentiation in monocytes.

### TLR binding and cytokine ELISA assay

After plating cells as described above, non-adherent cells were removed, and adherent cells were stimulated with either LPS (100 ng/mL) or **AVR-25** at different concentrations (10 µM for TLR4/TLR2 screening assay; 8, 80, 160 µM for TLR4, TNF-α and IL-6 assays; 0.8, 8.0, 80 µM for IL-10 assay; and 2.0, 4.0, 8.0, 16.0, 32.1, 62.2, 125, and-250 µM for the dose response study). For the TLR4, TLR2, and IL-10 assays, cells were treated only with **AVR-25**. After 48 h of incubation, cells were lysed and assayed for TLR4 and TLR2 while the supernatant was assayed for IL-10 protein levels using ELISA. For TNF-α, IL-1β and IL-6 assays, cells were treated with LPS followed by varying doses of **AVR-25** for 48 h, and the supernatant was assessed using ELISA (Raybiotech). For serum, i-NOS, CRP and MIP-1 were detected by ELISA (Abcam) following the manufacturer’s instructions.

### FASTPatch® assay

The hERG channel study was conducted at Charles River Laboratories, Cleveland, OH. Briefly, *in vitro* effects of compound **AVR-25** on the hERG (human ether-à-go-go-related gene) potassium channel current (a surrogate for IKr, the rapidly activating, delayed rectifier cardiac potassium current) expressed in HEK293 cells were evaluated at room temperature using the QPatch HT® (Sophion Bioscience A/S, Denmark), an automatic parallel patch clamp system. The test articles were evaluated at 0.3, 3, 30, and 300 μM, with each concentration tested in three sets of cells (N = 3). The duration of exposure to each test article concentration was approximately 5 minutes. Cisapride (0.05 µM) was used as a positive control to confirm the sensitivity of the test system to hERG inhibition.

### Ames test

Ames test was conducted at Covance Inc, Harrogate, UK according to their screening protocol in the absence and presence of S-9^43^. Treatments of **AVR-25** (3.2, 10, 32, 100, 320, and 700 µg/well) were conducted in strains TA100 (*Salmonella typhimurium*) in the absence and presence of metabolic activation system S-9 (Molecular Toxicology Incorporated, USA) from male Sprague-Dawley rats induced with Aroclor 1254 added as a 5% mix to the test system. The cultures were incubated at 37 ± 1 °C to provide a working culture of approximately 108 to 109 cells/mL, which was confirmed by either viability plating or optical density (OD) assessment at 650 nm. Revertant counts per well and the mean number of revertants (per well) were calculated for each treatment and strain. Sodium azide (NaN_3_, 0.3 µg/well) was used as a positive control for the S-9 experiment and 2-Aminoanthracene (AAN, 0.4 µg/well) was used for the +S-9 experiment to activate mutagenesis.

### Bacteria colony forming unit (CFU) study

Blood was collected by tail vein bleeds from all animals at 6, 24, and 48 h (hours) post-CLP surgery, and the end point of 72 h samples were collected by cardiac exsanguination after euthanasia by carbon dioxide asphyxiation. Aliquots of blood were transferred to 96-well plates and 10-fold serially diluted in sterile 1X PBS. The diluted samples were spot-plated (8 μl) on TSA + 5% sheep blood agar and aerobically incubated at 37 °C for 24 h. Colonies were counted for each dilution spot to calculate the final CFU/mL blood for each animal at each time point.

### Statistical analysis

Statistical analyses were performed using one-way analysis of variance (ANOVA) followed by Tukey’s post hoc test for comparison between three or more groups using GraphPad Prism software (version 7, GraphPad Software, San Diego, CA, USA). Statistical significance was defined as p < 0.05. Mean values were expressed as mean ± SEM. The number of mice/groups varied from N = 5–15.

## Supplementary information

Supplementary figures and Tables

## Data Availability

All data generated or analyzed during this study are included in this published article (and its Supplementary Information files). The datasets generated during and/or analyzed during the current study are also available from the corresponding author by reasonable request.
