# Supplementary material for: Novel Chitohexaose Analog Protects Young and Aged mice from CLP Induced Polymicrobial Sepsis
Source: Sci Rep. 2019 Feb 27;9:2904. doi: 10.1038/s41598-019-38731-3 (PMC6393422; doi:10.1038/s41598-019-38731-3)
Supplement: Supplementary file 1 — Supplementary figures and Tables [file 41598_2019_38731_MOESM1_ESM.pdf]

Novel Chitohexaose Analog Protects Young and Aged mice from CLP Induced Polymicrobial Sepsis

Pragnya Das[a,1], Santosh K Panda[b,1], Beamon Agarwal[c], Sumita Behera[d,g], Syed M Ali[e], Mark E Pulse[f], Joseph S Solomkin[g], Steven M Opal[h], Vineet Bhandari[a] and Suchismita Acharya[d,i,1]*

[a]*Department of Pediatrics, Division of Neonatology, Drexel University School of Medicine, Philadelphia, PA 19102, USA*

[b] *School of Medicine, Washington University, St. Louis. MO 63110, USA*

[c]*GenomeRxUS, 1250 Providence Rd, Secane, PA 19018*

[d]*AyuVis Research Inc, 1120 South Freeway, Fort Worth, TX 76104, USA*

[e]*Department of Biotechnology, Jamia Millia Islamia, New Delhi 110025, India*

[f]*Preclinical Service, University of North Texas Health Science Center, Fort Worth, Texas 76107, USA*

[g]*University of Cincinnati College of Medicine, Cincinnati, OH 45267, USA*

[h]*The Warren Alpert Medical School, Brown University, RI 02905, USA*

[i]*Acceleration laboratory, University of North Texas Health Science Center, Fort Worth, Texas 76107, USA*

[1]*Authors contributed equally;* [*]*corresponding author; E mail: sacharya@ayuvis.com*

Supplemental data

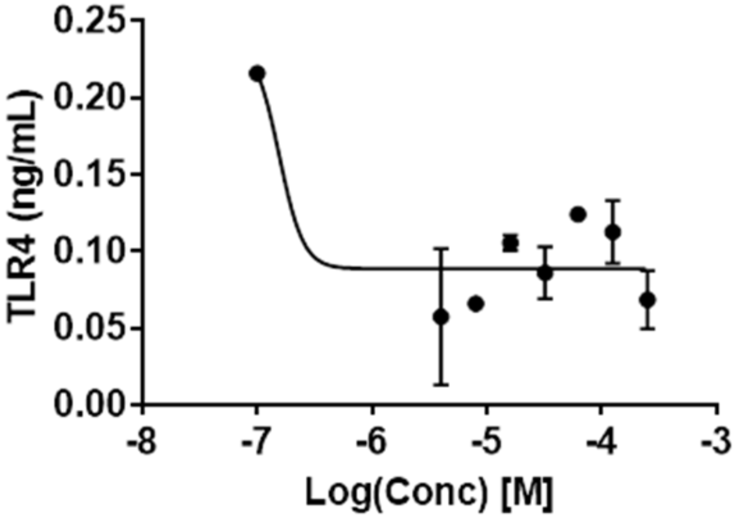

Fig. S1: Dose response study of **AVR-25** in THP-1 monocyte cells: $1x10^5$ THP cells (ATCC) seeded in 96 well plate was stimulated to macrophages with phorbol myristyl actetate (200 nM) for 48h and were treated with various concentrations (250µM- 2.0µM) of **AVR-25** serially diluted to half **(**in triplicates**)**. ELISA was performed after 48h of incubation on the cell lysate following the manufacturer instruction. IC_{50} of the **AVR-25** for TLR4 binding was calculated using GraphPad Prism7.04. (N = 3.) and found to be 0.156 µM.

S2

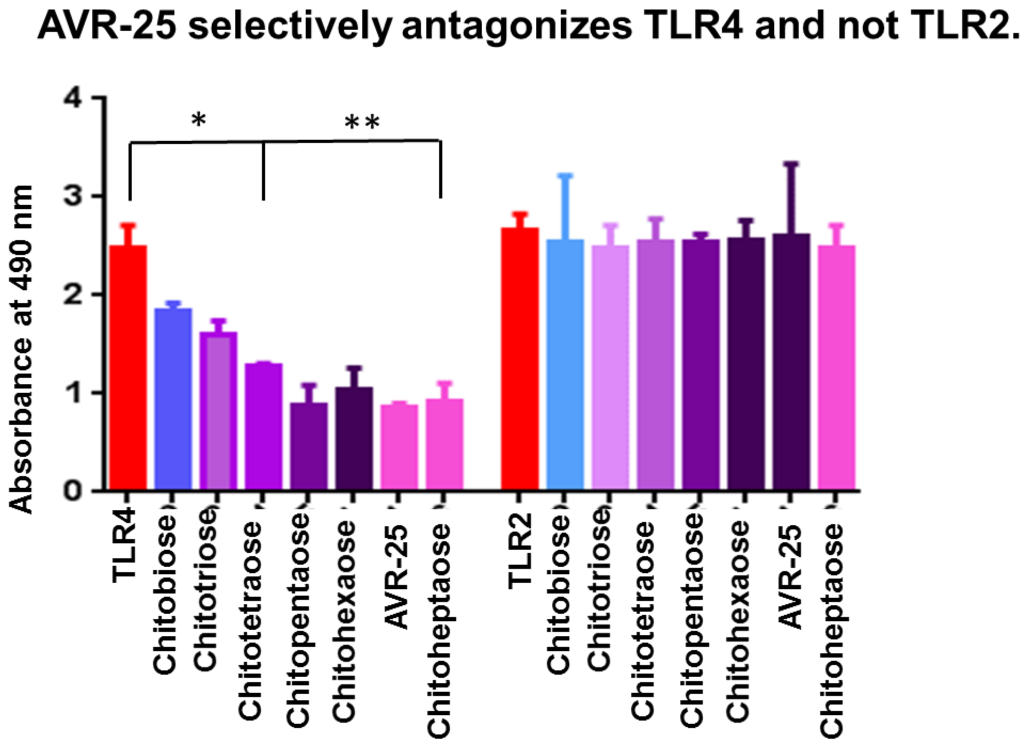

Fig. S2: Compound **AVR-25** binds to TLR4 but not to TLR2: Briefly, 0.5 million human PBMC (LeukoPak) were plated in 96 well plates and after 8–10h incubation at 37°C non-adherent cells were removed. Adherent cells were treated with 10µM of the compounds in PBS and incubated for 48h. TLR2 and TLR4 ELISA was performed on the cell lysate following the manufacturer's instruction. *p<0.01, **p<0.001 (N = 3).

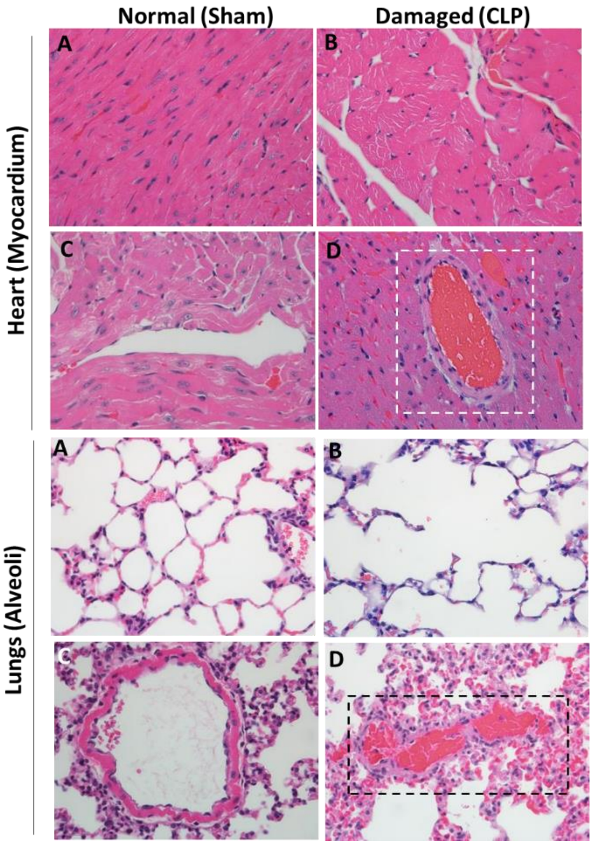
Normal (Sham)
Damaged (CLP)
Heart (Myocardium)
Lungs (Alveoli)
A
B
C
D

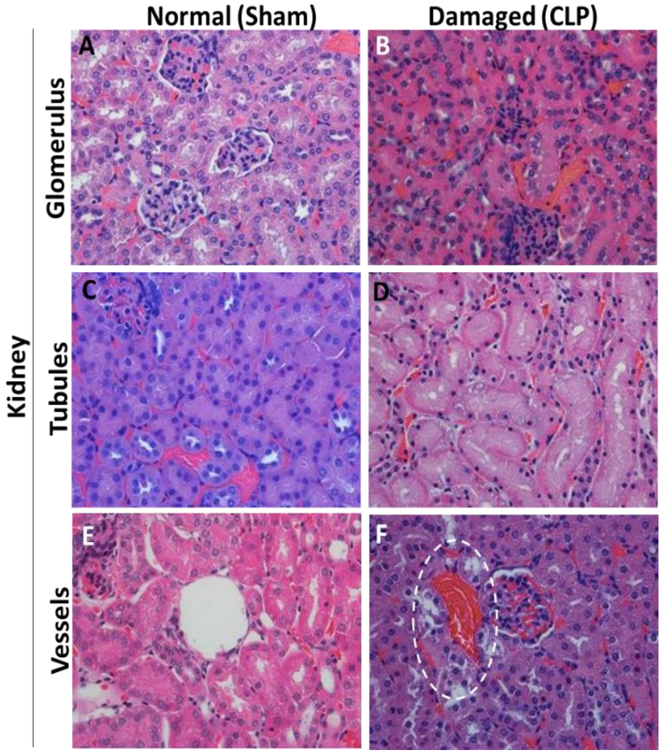
Normal (Sham)
Damaged (CLP)
Kidney
Glomerulus
Tubules
Vessels
A
B
C
D
E
F

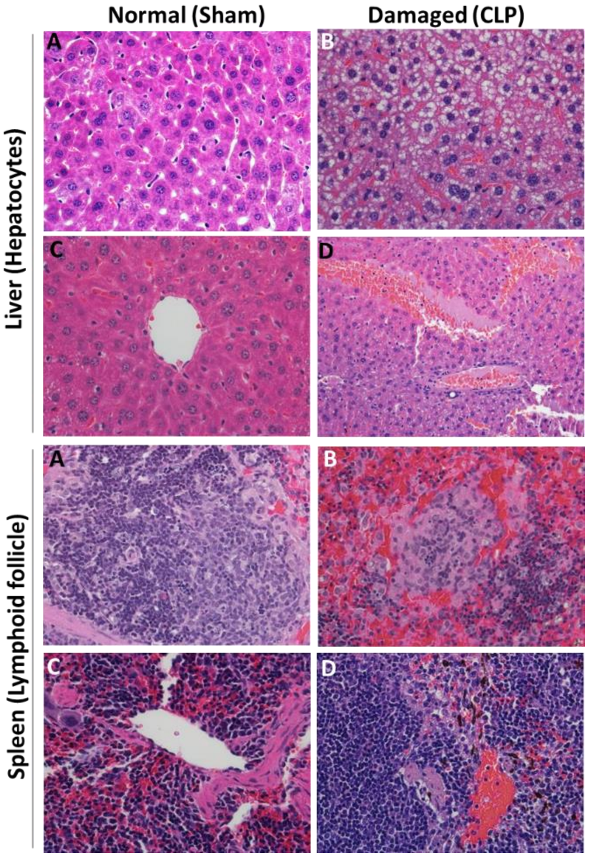
Normal (Sham)
Damaged (CLP)
Liver (Hepatocytes)
Spleen (Lymphoid follicle)
A
B
C
D

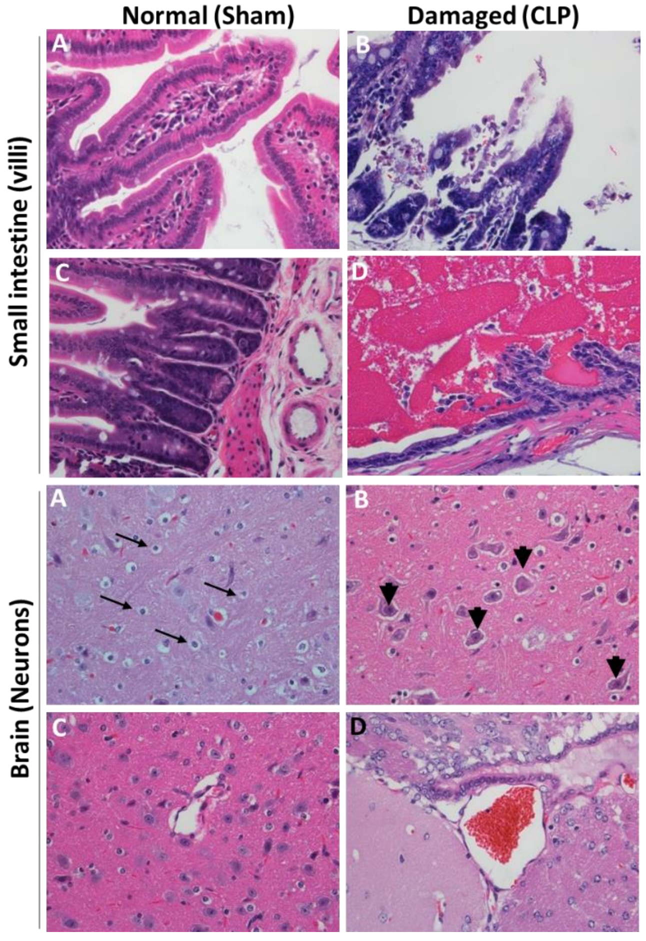
Normal (Sham)
Damaged (CLP)
Small intestine (villi)
Brain (Neurons)
A
B
C
D

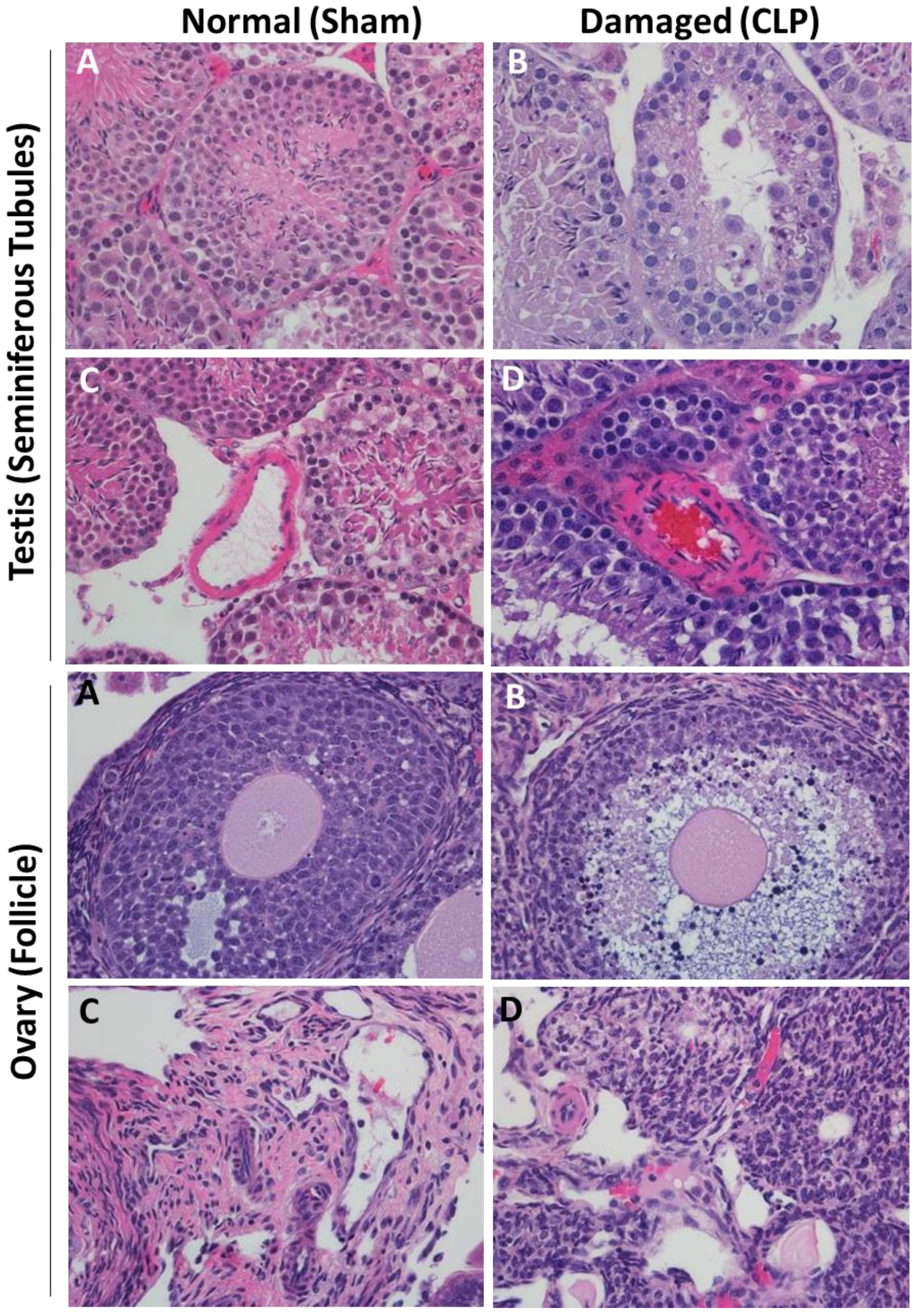

Fig. S3: Left panel shows normal (sham) while right panel shows damaged tissue following CLP procedure in mice. In the heart, the myocardium is damaged **(B)** and there is vascular congestion (dotted line; **D).** In the lungs, the alveolar membranes show fragmentation leading to expansion of sacs (**B)** with vascular congestion and hemorrhage (dotted line; **D).** In the liver, hepatocytic injury is characterized by cytoplasmic damage and variation in nuclear shape and size **(B)** and vascular congestion with hemorrhage **(D).** In the spleen, the lymphoid follicles show destruction **(B)**

associated with vascular congestion with hemorrhage **(D).** In the kidney, there is glomerular injury with reduction in size **(B)** and damaged tubules demonstrated by lack of nuclei **(D)** associated with vascular congestion with hemorrhage (dotted lines, **F**). In the small intestine, the villi show destruction **(B)** associated with vascular congestion with hemorrhage **(D).** In the brain, the neurons are injured as is evident from enlarged nuclei (arrowheads, **B**) as compared to normal nuclei (arrows, **A**). There are islands of vascular congestion and hemorrhage **(D)**. In the testis the seminiferous tubules are destroyed with lack of mature spermatocytes and spermatids **(B)** associated with vascular congestion with hemorrhage **(D).** In the ovary, the follicle show damage **(B)** and signs of vascular congestion and hemorrhage **(D).** In summary, CLP induces vascular congestion and hemorrhage in all the above organs, which is restored after treatment with AVR-25 as shown in **Figs. 5a, b**. (**X400**)

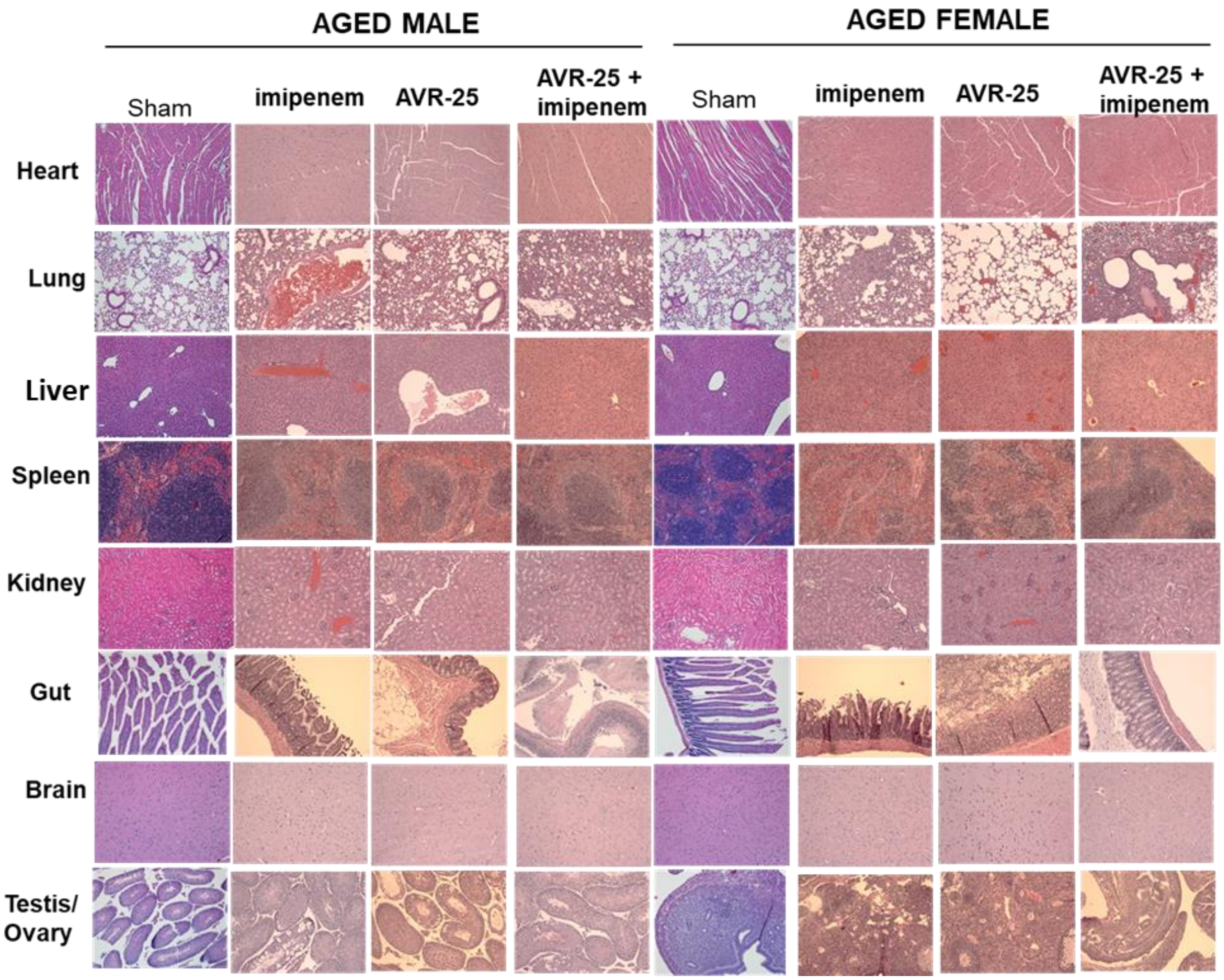

Fig. S4: Hematoxylin Eosin (H/E) of different organs of aged male and female CLP treated mice (10-12 weeks old) with **imipenem** only, **AVR-25 only** and **AVR-25 + imipenem**. **Imipenem** alone treated mice showed relatively less microthrombi and congestion in the heart, lungs, liver, kidney and brain, increased germinal centers size in spleen, necrosis of villi in gut and loss of testicular epithelium. On treatment with **AVR-25** all these changes showed further improvement to a major extent in both males and females, and tissues resembled the Sham group. CLP group not shown. (N =4-5). X100

Fig. S5

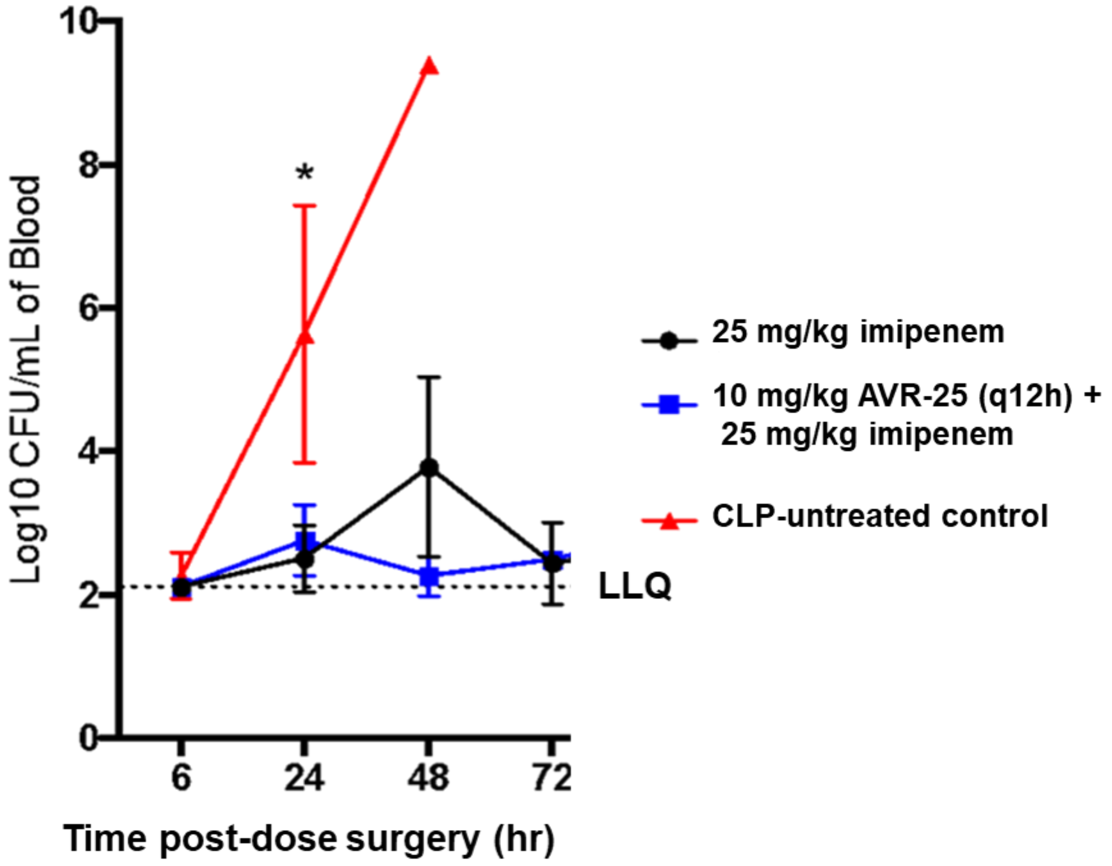

Fig. S5: Mean Log10 blood CFU from aged female mice study: The 24-hr mean log10 CFU values for the imipenem treated group and the **AVR-25 + imipenem** treated group were significantly different to the mean 24-hr log10 CFU for the untreated control group (N=8; p-value <0.05, multiple t tests comparison with Holm-Sidak post hoc analysis of significance).

Table S1: Calculated Mutagenicity Data (Ames assay)

Strain	Compound	Conc. Level (µg/well)	Fold Increase without S-9	Fold Increase with S-9
TA100	DMSO	-	-	-
	**AVR-25**	3.2	1.0	0.8
		10	1.0	1.1
		32	0.7	1.2
		100	1.1	1.1
		320	1.0	1.3
		700	1.0	1.1
	AAN	0.4	----	5.8
	NaN_3	0.3	6.4	----

Table S2: Effects of Test Articles on hERG Ion Channel Current

Test Article ID	IC_{50} (µM)	Conc (µM)	Mean % hERG Inhibition	Standard Deviation	n
**AVR-25**	>300	0.3	4.4	5.3	3
		3	7.4	7.7	3
		30	13.1	4.0	3
		300	15.2	2.7	3
Cisapride		0.05	62.3	1.9	3

Table S3:

Histology scoring was performed for each individual tissue. The following cells in each of the organs was scored on the total percentage of area damage: Heart (Myocardium), Lung alveoli, Kidney (glomeruli, tubules), Spleen (lymphoid follicles), Liver (Hepatocyte), Brain (Neuronal Damage), Testis (Seminiferous tubules) and Ovary (Follicles). Vascular congestion and hemorrhage were assessed by the number of areas/High power field (HPF). The following scoring system was followed:

**Tissue**	**Score**					
	**0**	**1**	**2**	**3**	**4**	**5**
**Heart**						
Myocardial Damage %	0	0-20	21-40	41-60	61-80	81-100
Vascular Congestion & Hemorrhage (#/HPF)	0	1	2	3	4	5
**Lung**						
Alveolar Damage %	0	0-20	21-40	41-60	61-80	81-100
Vascular Congestion & Hemorrhage (#/HPF)	0	1	2	3	4	5
**Liver**						
Hepatocyte Injury %	0	0-20	21-40	41-60	61-80	81-100
Vascular Congestion and Hemorrhage (#/HPF)	0	1	2	3	4	5
**Spleen**						
Lymphoid Follicles Damage %	0	0-20	21-40	41-60	61-80	81-100
Vascular Congestion & Hemorrhage (#/HPF)	0	1	2	3	4	5
**Kidney**						
Glomeruli Injury %	0	0-20	21-40	41-60	61-80	81-100
Tubular Damage %	0	0-20	21-40	41-60	61-80	81-100
Vascular Congestion & Hemorrhage (#/HPF)	0	1	2	3	4	5
**Gut**						

Mucosal Villi Damage %	0	0-20	21-40	41-60	61-80	81-100
Vascular Congestion & Hemorrhage (#/HPF)	0	1	2	3	4	5
**Brain**						
Neuronal Damage %	0	0-20	21-40	41-60	61-80	81-100
Vascular Congestion & Hemorrhage (#/HPF)	0	1	2	3	4	5
**Testis**						
Tubular Epithelial Damage %	0	0-20	21-40	41-60	61-80	81-100
Vascular Congestion & Hemorrhage (#/HPF)	0	1	2	3	4	5
**Ovary**						
Ovarian Follicular damage %	0	0-20	21-40	41-60	61-80	81-100
Vascular Congestion & Hemorrhage (#/HPF)	0	1	2	3	4	5

The summary of the scores were in each of the male mice individual groups were:

**Tissue**	**Male Sham**	**Male CLP**	**Male CLP +Imipenem**	**Male CLP +AVR-25**	**Male CLP +Imipenem + AVR-25**
**Heart**					
Myocardial Damage	0	4.7 ± 0.12	3.5 ± 0.13	3.5 ± 0.13	0.6 ± 0.13
Vascular Congestion & Hemorrhage	0	4.7 ± 0.12	3.6 ± 0.13	3.3 ± 0.18	0.66 ± 0.13
**Lung**					
Alveolar Damage	0	4.7 ± 0.12	3.4 ± 0.16	3.3 ± 0.19	0.53 ± 0.13
Vascular Congestion & Hemorrhage	0	4.6 ± 0.13	3.5 ± 0.13	3.5 ± 0.13	0.53 ± 0.13
**Liver**					
Hepatocyte Injury	0	4.6 ± 0.13	3.5 ± 0.13	3.5 ± 0.13	0.4 ± 0.13
Vascular Congestion & Hemorrhage	0	4.7 ± 0.12	3.3 ± 0.18	3.7 ± 0.13	0.6 ± 0.13
**Spleen**					

Lymphoid Follicles Damage	0	4.7 ± 0.11	3.6 ± 0.12	3.4 ± 0.17	0.6 ± 0.13
Vascular Congestion & Hemorrhage	0	4.7 ± 0.12	3.5 ± 0.13	3.3 ± 0.17	0.6 ± 0.13
**Kidney**					
Glomeruli Injury	0	4.5 ± 0.13	3.6 ± 0.13	3.5 ± 0.13	0.6 ± 0.13
Tubular Damage	0	4.7 ± 0.12	3.4 ± 0.16	3.5 ± 0.2	0.46 ± 0.13
Vascular Congestion & Hemorrhage	0	4.8 ± 0.1	3.4 ± 0.12	3.6 ± 0.13	0.6 ± 0.13
**Gut**					
Mucosal Villi Damage	0	4.6 ± 0.13	3.2 ± 0.18	3.3 ± 0.16	0.46 ± 0.13
Vascular Congestion & Hemorrhage	0	4.6 ± 0.13	3.3 ± 0.18	3.2 ± 0.18	0.6 ± 0.13
**Brain**					
Neuronal Damage	0	4.4 ± 0.17	3.3 ± 0.18	3.2 ± 0.19	0.8 ± 0.15
Vascular Congestion & Hemorrhage	0	4.6 ± 0.13	3.1 ± 0.2	3.1 ± 0.19	0.5 ± 0.14
**Testis**					
Tubular Epithelial Damage	0	4.7 ± 0.12	3.4 ± 0.19	3.4 ± 0.17	0.33 ± 0.12
Vascular Congestion & Hemorrhage	0	4.8 ± 0.11	3.5 ± 0.13	3.5 ± 0.13	0.73 ± 0.19

The summary of the scores were in each of the female mice individual groups were:

Tissue	Female Sham	Female CLP	Female CLP +Imipenem	Female CLP +AVR-25	Female CLP +Imipenem + AVR-25
**Heart**					
Myocardial Damage	0	4.8 ± 0.11	3.5 ± 0.13	3.5 ± 0.13	0.7 ± 0.19
Vascular Congestion & Hemorrhage	0	4.7 ± 0.12	3.4 ± 0.19	3.4 ± 0.17	0.3 ± 0.12
**Lung**					
Alveolar Damage	0	4.6 ± 0.22	3.2 ± 0.2	3.1 ± 0.18	0.4 ± 0.13
Vascular Congestion & Hemorrhage	0	4.5 ± 0.17	3.3 ± 0.18	3.2 ± 0.18	0.7 ± 0.16

**Liver**					
Hepatocyte Injury	0	4.8 ± 0.11	3.3 ± 0.19	3.2 ± 0.18	0.5 ± 0.13
Vascular Congestion & Hemorrhage	0	4.7 ± 0.12	3.2 ± 0.18	3.3 ± 0.16	0.5 ± 0.13
**Spleen**					
Lymphoid Follicles Damage	0	4.7 ± 0.12	3.5 ± 0.13	3.6 ± 0.13	0.6 ± 0.13
Vascular Congestion & Hemorrhage	0	4.7 ± 0.12	3.5 ± 0.13	3.3 ± 0.17	0.6 ± 0.13
**Kidney**					
Glomeruli Injury	0	4.7 ± 0.13	3.5 ± 0.13	3.5 ± 0.13	0.5 ± 0.13
Tubular Damage	0	4.9 ± 0.09	3.5 ± 0.17	3.3 ± 0.19	0.6 ± 0.13
Vascular Congestion & Hemorrhage	0	4.7 ± 0.11	3.7 ± 0.13	3.4 ± 0.17	0.6 ± 0.13
**Gut**					
Mucosal Villi Damage	0	4.7 ± 0.12	3.3 ± 0.19	3.6 ± 0.13	0.6 ± 0.13
Vascular Congestion & Hemorrhage	0	4.8 ± 0.11	3.5 ± 0.13	3.5 ± 0.13	0.4 ± 0.13
**Brain**					
Neuronal Damage	0	4.7 ± 0.12	3.5 ± 0.13	3.5 ± 0.13	0.5 ± 0.13
Vascular Congestion & Hemorrhage	0	4.7 ± 0.12	3.4 ± 0.16	3.3 ± 0.19	0.5 ± 0.13
**Ovary**					
Ovarian Follicular damage	0	4.7 ± 0.12	3.6 ± 0.13	3.3 ± 0.19	0.7 ± 0.13
Vascular Congestion & Hemorrhage	0	4.7 ± 0.13	3.5 ± 0.13	3.7 ± 0.12	0.6 ± 0.13
